# Professional calling among nursing students: a latent profile analysis

**DOI:** 10.1186/s12912-023-01470-y

**Published:** 2023-09-02

**Authors:** Hu Jiang, Yongxia Mei, Xiaoxuan Wang, Zhixin Zhao, Beilei Lin, Wenna Wang, Zhenxiang Zhang

**Affiliations:** 1 School of Nursing and Health, Zhengzhou University, Henan, China; 2 Nursing department, The Third Affiliated Hospital of Zunyi Medical University (The First People’s Hospital of Zunyi), Zunyi, China

**Keywords:** Calling, Nursing, Student, Latent profile analysis, Influencing factors

## Abstract

**Background:**

One factor that influences nursing students' decision to pursue a nursing career is professional calling. It is important to comprehend nursing students' professional calling, which may have an impact on their career choice and career development.

**Objectives:**

To investigate possible calling types and contributing variables among nursing students.

**Design:**

Cross-sectional descriptive study.

**Participants:**

A total of 10,583 nursing students were enrolled in this survey.

**Methods:**

From November 16th, 2022, to January 17th, 2023, a cross-sectional study was carried out among nursing students using a convenient sampling. The subjects were given the Chinese Calling Scale and the General Demographic Information Questionnaire. Latent profile analysis (LPA) was used to separate nursing students' professional calling into a variety of subgroups. To find the variables connected to the prospective calling categories, we used ordinal and multinomial Logistic regression analysis.

**Results:**

Respondents were divided into three calling groups, low (*N* = 3204), moderate (*N* = 4492), and high calling group (*N* = 2887), which accounted for 30.3%, 42.4%, and 27.3% of the total respondents, respectively, in accordance with the findings of the latent profile analysis. Across scale scores and dimensions for the three separate categories, three groups demonstrated statistically significant differences (both *p* < 0.001). Profile membership was predicted by 8 factors such as age, gender, location of origin, first volunteer experience, highest degree earned, marital status, student leadership experience, and political appearance.

**Conclusion:**

Three latent calling patterns were found, and there was calling variability across nursing students. Special care should be given to students with low calling. Nursing students must use professional education tools to help them develop their career calling and stabilize the nursing team.

## Introduction

The healthcare system is facing a critical shortage of nurses in the globe for high levels of work-related stress, burnout, and turnover rates, as well as their own ageing [1–3]. Yet, the gradual drop in nursing students, especially top-notch students, choosing to pursue a nursing profession has made the shortage even worse.

There are many different and complicated reasons why nursing students give up on their careers in nursing. Students' decisions to enter, continue in, or leave the nursing profession have been connected to perceptions of nursing [4, 5]. It has been reported that 17.6% of new nurses intend to leave their positions within the first two years of employment [6]. A growing body of research has linked calling and positive work outcomes in recent years as a result of positive psychology. A nurse who is "called" to the nursing profession will be more likely to remain in the profession. However, little of this research has been presented to nursing students.

The definition of calling has not yet been agreed upon by researchers. A transcendent summon to approach a particular life role in a way focused on demonstrating or deriving a sense of purpose or meaningfulness and that prioritizes other-oriented values and goals as the main sources of motivation is known as a "calling," and it is experienced as coming from beyond the self [7]. A sense of calling can act as a motivating factor, a sense of personal fit, or an altruistic motivation, according to a qualitative research [8]. Emerson [9] described it as a calling is a passionate intrinsic drive or desire to serve others via involvement in nursing practice as a way to give one's life meaning, in accordance with the qualities of the nursing discipline.

The function of calling among nurses has been investigated and understood. Nursing professionals who are called see their work as a meaningful mission rather than a way to make money, which inspires them to participate [9, 10]. Calling has been demonstrated to promote nurses' work motivation, job happiness, and engagement as well as provide them the confidence to handle pressure at the workplace and provide higher-quality patient care [11–14]. The research that is currently available indicates that calling is vital important to nurses.

Nursing students' key decisions and professional aspirations are significantly influenced by their calling. Students viewed nursing as both an option and a calling, according to Miller [15]. A longitudinal study found a significant positive correlation between calling and career preparation [16]. It has also been shown in other studies that there is a connection between calling, professional identity [17], and career commitment [18] among college students.

The Career Construction Theory (CCT) stands as the theoretical framework with which our study is formulated [19]. Career calling, according to CCT, is a dynamic construct that develops throughout a person's life before they begin working, and affected by many internal and external factors [20, 21]. The concept of professional calling refers to aligning one's career with a strong sense of purpose, which is reflected in actions, thoughts, and emotions vital to achieving career goals. There is still room for further investigation into the current status of nursing students' professional callings, as well as whether there is heterogeneity within their groups.

Studies on calling among nursing students are generally cross-sectional, do not analyze calling in depth, and lack a significant sample size. Latent profile analysis (LPA) is a person-centered screening and classification approach that categorizes samples according to various individual traits or factors and homogenous grouping of continuum data that can separate groups with comparable symptoms into subgroups [22]. In other words, potential profile analysis is a statistical technique that uses potential variables to explain the relationship between exogenous continuous-type indicators, allowing for the estimation of the association between exogenous indicators and the maintenance of local independence among exogenous indicators. LPA's central tenet is that the probability distributions of the exogenous variable responses may be described by a small number of latent variables that are mutually exclusive and each have a unique propensity to choose responses to the exogenous variables [23]. In psychology research, this method is frequently used to count the number of subpopulations in a sample. Also, because LPA needs a sample size larger than 500, it is appropriate for investigations with big samples [24].

There is an urgent need to further examine the present situation and probable antecedents of calling among nursing students in the wake of the COVID-19 outbreak, even though research have reported on the current status and perceptions of calling among nursing students. In order to analyze nursing students' calling, assess the contributing elements, and develop prospective categories, this study performed a cross-sectional survey of nursing students.

## Methods

### Study design

In this study, an online survey was used to conduct a cross-sectional analysis. The design and reporting of this study were conducted in accordance with the guidelines for Strengthening the Reporting of Observational Studies in Epidemiology (STROBE).

### Settings

This study was primarily designed for nursing schools in China.

### Participants

All nursing students who met the inclusion requirements for the study were invited to take part in the optional survey. The following criteria have to be met in order to be eligible to take part in this study: 16-year-olds who are full-time students, have access to the internet, have full cognitive and behavioral competence, give informed consent, and participate voluntarily in the survey. The pursuing exclusion criteria were applied: diagnosed with a serious mental disease or psychiatric condition; on leave from school.

### Sample size

LPA requires a sample size greater than 500 [24], so the sample size for this study is at least 500.

### Data collection tools

Based on the literature and the study's goal, the authors created a generic information questionnaire to collect information in general. Age, gender, ethnicity, place of birth, first volunteer experience, highest degree, education level, marital status, student leader experience, and political appearances were among the demographic and sociological factors that were crucial to the study.

The Chinese Calling Scale was developed by Chunyu Zhang [25], which consists of 11 items. It uses a 5-point Likert scale from “very non-conforming” to “very conforming” to measure three dimensions of career orientation: altruism, guiding force, and meaning and purpose. Altruism is the aspiration to benefit society through work, while guiding force is the drive for career advancement. Meaning and purpose analyze the link between career and life’s significance. The total score range is 11–55. The high internal consistency and reliability of the scale is indicated by a Cronbach’s alpha value of 0.882. The scale has been proven to be valid and reliable for use in assessing career orientation among Chinese nurse and nursing students.

### Data collection

The information was gathered during November 16th, 2022, to January 17th, 2023. To recruit study participants, the project leader contacted the nursing school's director from different levels of schools across the country and distributed the web-based questionnaire to the students through the director. Wenjuanxing (www.wjx.cn), a popular online data gathering tool in China, was utilized to produce a web survey. WeChat, a well-known social media platform, was used to distribute both the QR code and link to the online questionnaire. Each IP address can only be entered once to avoid duplication.

### Statistical analyses

The results of the 11 items on the Chinese calling scale served as exogenous variables, due to the scale scores were continuous variables, and a latent profile analysis was carried out using the Mplus 7.4 program to separate nursing students' professional calling into a variety of subgroups. The three fit indicators in the following categories were used to assess model fit and determine the appropriate number of categories: (1) The lower the value for Log likelihood (LL), Akaike information criterion (AIC), Bayesian information criterion (BIC) and the Adjusted Bayesian Information Criteria (aBIC), the better the model fit. (2) Entropy, the closer a number is to 1, the more accurate the categorization. (3) Lo-Mendell-Rubin (LMR), and Bootstrapped Likelihood Ratio Test (BLRT). LMR and BLRT show significant* p*-values when evaluating the goodness of fit differences between k potential class and k-1 potential class. The k- potential class outperformed BLRT when linked with significant *p*-values (*p* < 0.05), which was the case. The models from each class's fitting results were mixed with the aforementioned indications to determine which model suited the data the best.

The SPSS 26.0 statistical program was used to analyze the data. We used frequency and composition ratios to describe categorical variables, while mean and standard deviation were used to describe continuous variables if they passed the normality test. In order to compare categorical variables between groups, the chi-square test was performed. Continuous variables were compared between groups using an ANOVA. When significant between-group effects were observed, post hoc analysis were performed using the Tukey HSD method. A multivariate logistic regression model was used to evaluate the differences in demographic characteristics and employment factors among nurses with various types of occupational perks.* p* < 0.05 was used to indicate that a difference was statistically significant.

### Ethical considerations

The Zhengzhou University Ethics Committee gave its approval to this study, and the subjects gave their consent before their data was collected. Participants were given information about the study's major points and motivations before to beginning the survey, at which point they can decide whether to offer informed consent. The survey collection was shut down automatically if the participant rejected to take part. The fact that the study's findings would be released in aggregate form and that survey respondents couldn't be personally identified was also disclosed to participants.

## Results

### Participant characteristics

This study included 10,756 nursing students in total, and with a final valid return rate of 98.4%, 173 questionnaires had missing information. The students ranged in age from 17 to 40, with a mean age of 19.51 (SD 1.83). 9045 women made up 85.5% of the population, with 1538 men making up the remaining 15.5%. 622 (5.9%) were members of ethnic minorities, while 9961 (94.1%) were Han Chinese. In addition, 2213 people (20.9%) came from metropolitan regions, making up 8370 (79.1%) from urban or rural areas. The results were shown in Table 1.

**Table 1:** General information of the participants (*N* = 10,583)

Variables	Mean (SD)	n (%)
Age	19.51 (1.83)	
Gender		
Male		1538 (14.5)
Female		9045 (85.5)
Ethnic group		
Han Chinese		9961 (94.1)
Ethnic Minority		622 (5.9)
Place of origin		
Rural		8370 (79.1)
Urban		2213 (20.9)
First volunteer		
Yes		6225 (58.8)
No		4358 (41.2)
Highest Degree		
Junior college		8459 (79.9)
Bachelor’s		1886 (17.8)
Master’s and above		238 (2.3)
Education Level		
Senior college		8238 (77.8)
General Undergraduate		2107 (19.9)
Double-class institutions		238 (2.3)
Marital status		
Single/Divorced		10450 (98.7)
Married		133 (1.3)
Student leader experience		
Yes		3393 (32.1)
No		7190 (67.9)
Political Appearance		
Member of the Communist Youth League		323 (3.1)
Preparatory member or member of the Communist Party of China		5679 (53.7)
General public		4530 (42.8)
Others		51 (0.5)

### Characteristics of the different classes

**Fig. 1:**
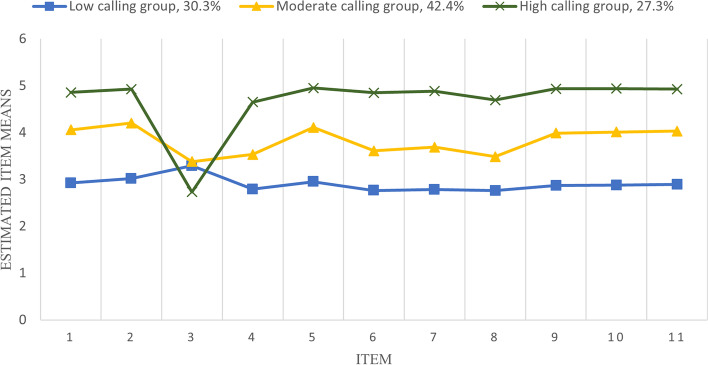
Latent profiles of calling

Several profiles were developed based on the Chinese calling scale evaluation findings. The LMR and BLRT tests were significant, and Class 4 had the lowest BIC value and the closest Entropy value to 1. Nevertheless, just 3.1% of the total is accounted for by one categorization, which is not statistically significant. Class 3, which contained three potential profiles, was determined to be the model with the best fit indices when the models' fit indices were evaluated. The average chance of nurses falling into each group was 100%, making the model results believable. Table 2 presented the outcomes. The Class 1, Class 2, and Class 3 were designated in accordance with their distinctive distribution based on the mean scores of each class in the impression of calling as calculated using Class 3 and displayed in Fig. 1. The Class 3 had the highest overall score, although the third item has the lowest score, but this item is reverse scored, and a lower score indicates a higher calling score, then the C3 was categorized as "high calling group" (27.3%), Class 2 was labeled as "moderate calling group" (42.4%), and Class 1 was labeled as "low calling group" (30.3%). For each of the three distinct subgroups, further analysis revealed particular statistically significant variations in scale scores and dimensions (both *p* < 0.001). A post hoc analysis using the Turkey HSD method found that nursing students in the high calling group scored higher than the moderate and low calling group on both the total scale score and each dimension. The moderate calling group also scored higher than the low calling group. Table 3 presented the outcomes.

**Table 2:** Potential profile analysis indicators (*N* = 10,583)

Model	LL^a^	AIC^b^	BIC^c^	aBIC^d^	Entropy	LMR^e^ *p*-value	BLRT^f^ *p*-value	Category probability (%)
Class 1	-165,389.470	330,822.939	330,982.813	330,912.900	-	-	-	-
Class 2	-137,598.513	275,265.026	275,512.105	275,404.057	0.941	< 0.001	< 0.001	42.2/57.8
Class 3	-124,950.988	249,993.975	250,328.257	250,182.075	0.954	< 0.001	< 0.001	30.3/42.4/27.3
Class 4	-114,625.504	229,367.008	229,788.495	229,604.179	0.968	< 0.001	< 0.001	3.1/39.3/30.3/27.3

^a^ Log likelihood, ^b^ Akaike information criterion, ^c^ Bayesian information criterion, ^d^ Adjusted bayesian information criterion, ^e^ Lo-Mendell-Rubin Likelihood Ratio Test, ^f^ Bootstrapped Likelihood Ratio Test

**Table 3:** Calling scores and dimensions in different categories (*N* = 10,583)

Variables	Class 1 Mean (SD)	Class 2 Mean (SD)	Class 3 Mean (SD)	*F*	*p*
Calling	31.91 (4.81)	42.09 (2.84)	51.37 (2.12)	24,787.80	< 0.001
Altruism	12.16 (1.81)	15.76 (2.01)	17.47 (1.90)	6195.99	< 0.001
Guiding Force	11.10 (2.30)	14.30 (2.10)	19.09 (1.49)	11,991.99	< 0.001
Meaning and Purpose	8.64 (1.75)	12.03 (1.25)	14.81 (0.61)	17,228.40	< 0.001

### Demographic and related characteristics of each profile

In the univariate analysis, the three latent profiles were impacted by characteristics such as age, gender, location of origin, first volunteer experience, highest degree, education level, grade, marital status, student leader experience, and political appearance (both *p* < 0.05). The outcomes were displayed in Table 4.

**Table 4:** Demographic and characteristics by latent profile (*N* = 10,583)

Variables	Class 1	Class 2	Class 3	χ^2^/*F*	*p*
Age, Mean (SD)	19.45 (1.57)	19.51 (1.90)	19.60 (1.96)	4.688	0.009
Gender				61.94	< 0.001
Male	525 (16.4%)	513 (11.4%)	500 (17.3%)		
Female	2679 (83.6%)	3979 (88.6%)	2387 (82.7%)		
Ethnic group					
Han Chinese	3017 (94.2%)	4241 (94.4%)	2703 (93.6%)	1.975	0.372
Ethnic Minority	187 (5.8%)	251 (5.6%)	184 (6.4%)		
Place of origin				10.988	0.004
Rural	2583 (80.6%)	3559 (79.2%)	2228 (77.2%)		
Urban	621 (19.4%)	933 (20.8%)	659 (22.8%)		
First volunteer				21.595	< 0.001
Yes	1967 (61.4%)	2530 (56.3%)	1728 (59.9%)		
No	1237 (38.6%)	1962 (43.7%)	1159 (40.1%)		
Highest Degree				95.222	< 0.001
Junior college	2725 (80.0%)	3428 (76.3%)	2306 (79.9%)		
Bachelor’s	442 (13.8%)	935 (20.8%)	509 (17.6%)		
Master’s and above	37 (1.2%)	129 (2.9%)	72 (2.5%)		
Education Level				72.623	< 0.001
Senior college	2650 (82.7%)	3351 (74.6%)	2237 (77.5%)		
General Undergraduate	504 (15.7%)	1019 (22.7%)	584 (20.2%)		
Double-class institutions	50 (1.6%)	122 (2.7%)	66 (2.3%)		
Marital status				6.455	0.040
Single/Divorced	3177 (99.2%)	4429 (98.6%)	2844 (98.5%)		
Married	27 (0.8%)	63 (1.4%)	43 (1.5%)		
Student leader experience				76.459	< 0.001
Yes	889 (27.7%)	1405 (31.3%)	1099 (38.1%)		
No	2315 (72.3%)	3087 (68.7%)	1788 (61.9%)		
Political Appearance				44.999	< 0.001
Member of the Communist Youth League	1628 (50.8%)	2448 (54.5%)	1603 (55.5%)		
Preparatory member or member of the Communist Party of China	69 (2.2%)	157 (3.5%)	97 (3.4%)		
General public	1480 (46.2%)	1847 (41.7%)	1176 (40.7%)		
Others	27 (0.8%)	13 (0.3%)	11 (0.4%)		

A multinomial logistic regression analysis was carried out using class 1 as the reference group in order to pinpoint the variables connected to calling among the three profiles. The outcomes were displayed in Table 5. The findings showed that age was a predictor of class 2 and was associated with a reduced sense of calling. Men were underrepresented in Class 2. Rural students and those who were single, or divorced had a higher likelihood of being placed in Class 2 (*OR*: 0.539, 95%*CI*: 0.320–0.907). In Class 3, there were fewer pupils who resided in rural regions (*OR*: 0.864, 95%*CI*: 0.762–0.980). Junior college is a consistent predictor and protective factor for all profiles among the highest degrees. The lowest percentage in Class 2 is for bachelor's degrees (*OR*: 0.521, 95%*CI*: 0.335–0.810). These three traits share a similar indicator: prior experience as a student leader. Political Appearance frequently predicted Class 2 and Class 3.

**Table 5:** Predictors of latent profile membership

Variables	Class 1 VS Class 2			Class 1 VS Class 3		
	***β***	***OR***	**95%*CI***	***β***	***OR***	**95%*CI***
Age	-0.058	0.944**	0.912–0.976	-0.001	0.999	0.963–1.036
Gender						
Male	-0.416	0.659***	0.577–0.753	0.030	1.030	0.898–1.181
Female		Ref			Ref	
Marital status						
Single/Divorced	-0.618	0.539*	0.320–0.907	-0.440	0.644	0.367–1.130
Married		Ref			Ref	
Place of origin						
Rural	-0.032	0.968	0.862–1.087	-0.146	0.864*	0.762–0.980
Urban		Ref			Ref	
First volunteer						
Yes	-0.181	0.834***	0.760–0.916	-0.056	0.945	0.852–1.049
No		Ref			Ref	
Education Level						
Senior college	0.071	1.074	0.687–1.677	-0.024	0.976	0.601–0.922
General Undergraduate	-0.055	0.947	0.655–1.369	0.058	1.060	0.705–1.593
Double-class institutions		Ref			Ref	
Highest Degree						
Junior college	-1.270	0.281***	0.167–0.472	-0.577	0.561*	0.321–0.982
Bachelor’s	-0.652	0.521**	0.335–0.810	-0.422	0.656	0.406–1.059
Master’s and above		Ref			Ref	
Student leader experience						
Yes	0.158	1.171**	1.057–1.298	0.431	1.538***	1.377–1.718
No		Ref			Ref	
Political Appearance						
Member of the Communist Youth League	1.062	2.893**	1.482–5.647	0.856	2.354*	1.158–4.783
Preparatory member or member of the Communist Party of China	1.173	3.231**	1.554–6.717	0.849	2.336*	1.072–5.090
General public	1.004	2.729**	1.397–5.331	0.731	2.077*	1.021–4.226
Others		Ref			Ref	

*OR *Odds ratio, 95% *CI *95% Confidence Interval, *Ref *Refer

^*^p < 0.05, ** *p* < 0.01, *** *p* < 0.001

## Discussion

This is, as far as we are aware, the first study to look into the co-occurrence of different types of callings among nursing students. The current study examined the latent profiles of the calling and the factors that affect its connection based on the body of existing literature. The sample was split into three latent profiles based on indicator parameters. The research showed that calling elicited distinct responses from nursing students. Latent profiles also showed significant features in terms of research aspects.

The profiles were characterized as high, moderate, and low calling group. Each group’s average score was 51.37, 42.09, and 31.91, respectively. The results were similar to another Chinese research [26], which indicated that the average score of calling among nursing students was 41.17. The findings revealed that most students had positive views of the nursing profession, which were in line with findings from earlier research [17, 27]. However, the low calling group accounting for 30.3% of the total participants, which revealed that nurses' calling was lower than that of other care professionals as a result of their subpar remuneration and working circumstances [28].

The three dimensions (altruism, guiding force, meaning and purpose) of calling are highly compatible with nursing's professional values. Nursing students were taught to be a nurse that focuses on helping and caring for others. Nursing students had a feeling of being called during the COVID-19 epidemic. Nursing students also have obtained good professional identification and professional values because of a favorable social image of nurses [29, 30]. Unfortunately, the epidemic did not elevate a portion of nurses' professional calling, and as a result, some nursing students left the profession [31]. In addition to professional calling, there is still a need to focus on this area.

Our research also indicated three profiles differed substantially in terms of their personal and social characteristics. Our findings demonstrated a negative relationship between age and calling, which is comparable to other research with a small exception. First- and fourth-year undergraduates had considerably greater levels of calling, according to Wang, et al. [32]. We suggest that the growth of age, improved professional awareness, and educational advancement may be responsible for the U-shaped developing pattern that is produced while calling in nursing students. Also, our research revealed that, to the largest degree, married status predicted profile membership, supporting the aforementioned viewpoint.

Because nursing is a predominantly female profession worldwide and there are very few male nurses, career confidence is lower [33, 34]. Yet since males play a significant role in the nursing industry, bringing in more of them might relieve the shortage of nurses while also enhancing the diversity and balance of the workforce [35].

The phenomenon of job choices between Eastern and Western nursing students are quite different. Chinese students with political backgrounds and prior student leadership roles could demonstrate greater commitment and responsibility. Religious traits were linked to a sense of calling among practicing primary care doctors and psychiatrists in the Western countries, it was discovered [36]. The majority of students decide to enroll in nursing school when they are still in high school or college, and most select nursing with a stronger feeling of calling [37]. Asia, however, operates entirely differently. According to a Korean study [38], rather than a person's decision or interest, the contextual backdrop and family advice had the most effects. The results of this study were comparable to earlier ones in that there was statistical relevance of place of origin among groups. The nursing profession is not very well-liked in China since nurses' social position and salaries are not very high. As a result, following the college admission exam, students frequently do not select a nursing vocation as their top option. Our study provides more evidence that calling and career choice are connected.

In this study, we provide empirical evidence for the antecedents of calling. The results of this study suggest that Career Construction Theory may provide an effective framework for exploring what factors contribute to nursing students' sense of calling. As to nursing students, college is an important stage in the development of a calling. Students with a higher nursing calling will be able to contribute more to a career as a nurse that is more stable and professional. More theoretical and practical insights are needed in this area in the future to explore the professional calling of nursing students.

Students should use their time in college to establish their professional identities and ideals. Although not everyone chooses to become a nurse, instructors, hospitals, society, and students' own viewpoints may all have an impact on their career decisions. A low degree of calling was discovered in 30.3% of nursing students, which calls for attention and help in this demographic. Programs that will aid nursing students in advancing their professions are now the subject of research. Han, et al. [39] found that their training gave nursing students a strong feeling of calling. Given the present absence of effective intervention programs, calling in nursing students is a crucial issue for future study. Our findings considerably add to the body of knowledge already in existence.

### Limitations

The research included a number of restrictions. First of all, even though we targeted to investigate nursing students across the country, but our study relied on a convenience-sample, self-reported web-based questionnaire, and the results may be biased. To increase the representativeness of the study sample in subsequent research, random sampling should be used. Additionally, some potential factors such as personal traits, educational performance, and family economic status were left out of the questionnaire, we also did not compare the score levels with other populations such as non-nursing medical students, general college students, and clinical nurses. Lastly, there were only nursing students from mainland China included in the study, and the results may vary slightly from those of other countries. Hence, more diverse populations, types, and rigor design studies are required to confirm the findings in the future.

## Conclusion

In the current study, we discovered three latent profiles and recognized the heterogeneity of calling among nursing students. Also, the social and personal calling factors were noted. Our findings lay the groundwork for the intervention programs and can be used to guide future research. Students with low calling should get specific attention among the three groupings. In order to enhance nursing students' career calling and so stabilize the nursing team, stakeholders should pay attention to professional education strategies in future research. Therefore, it is critical to work to improve policy settings, raise the social standing of the nursing profession, strengthen nursing professional education, and raise salaries for nursing staff, among other strategies, in order to promote the attractiveness of the nursing profession.

## Data Availability

The data supporting the findings of this study are available on request from the corresponding author. The data are not publicly available due to privacy or ethical restrictions.
